# Extended-Spectrum β-Lactamase-Producing Enterobacteriaceae in Hospitalized Neonatal Foals: Prevalence, Risk Factors for Shedding and Association with Infection

**DOI:** 10.3390/ani9090600

**Published:** 2019-08-23

**Authors:** Anat Shnaiderman-Torban, Yossi Paitan, Haia Arielly, Kira Kondratyeva, Sharon Tirosh-Levy, Gila Abells-Sutton, Shiri Navon-Venezia, Amir Steinman

**Affiliations:** 1 Koret School of Veterinary Medicine (KSVM), The Robert H. Smith Faculty of Agriculture, Food and Environment, The Hebrew University of Jerusalem, Rehovot 761001, Israel; 2 Department of Clinical Microbiology and Immunology, Sackler Faculty of Medicine, Tel Aviv University, Tel Aviv 6997801, Israel; 3 Clinical Microbiology Lab, Meir Medical Center, Kfar Saba 4428164, Israel; 4 Department of Molecular Biology, Faculty of Natural Science, Ariel University, Ariel 40700, Israel

**Keywords:** equine, foal, ESBL-E, antibiotic resistance, shedding, umbilical infection, risk factors

## Abstract

**Simple Summary:**

Multidrug-resistant (MDR) *Enterobacteriaceae* are becoming a major worldwide concern in human and veterinary medicine, mainly due to the production of extended-spectrum β-lactamases (ESBLs). These bacteria have been investigated in adult horses, but not in neonatal foals. In this study, we investigated extended-spectrum β-lactamase *Enterobacteriaceae* (ESBL-E) shedding and infection in hospitalized mares and their neonatal foals. Overall, we sampled rectal swabs from 55 pairs of mares and their foals on admission, and 33 of them were re-sampled on the 3rd day of hospitalization. We also collected clinical samples, when available. We found that shedding rates and bacterial species diversity increased significantly during hospitalization, both in mares and foals. On admission to hospital, foals’ shedding was associated with umbilical infection. During hospitalization, it was associated with ampicillin treatment. Foals’ shedding was independent of their mares’ shedding. Four foals were infected with ESBL-E strains, including umbilical infections and wounds. We suggest further investigation and surveillance of ESBL-E in neonatal foals, in order to reduce resistance rates and infections.

**Abstract:**

Extended-spectrum β-lactamase *Enterobacteriaceae* (ESBL-E) have been investigated in adult horses, but not in foals. We aimed to determine shedding and infection in neonatal foals and mares. Rectal swabs were sampled from mare and foal pairs on admission and on the 3rd day of hospitalization; enriched, plated, and bacteria were verified for ESBL production. Identification and antibiotic susceptibility profiles were determined (Vitek2). Genotyping was performed by multilocus sequence typing (MLST). Genes were identified by PCR and Sanger sequencing. Medical data were analyzed for risk factors (SPSS). On admission, 55 pairs were sampled, of which 33 pairs were re-sampled. Shedding rates on admission in foals and mares were 33% (95% CI 21–47%) and 16% (95% CI 8–29%), respectively, and during hospitalization, these increased significantly to 85% (95% CI 70–94%) and 58% (95% CI 40–73%), respectively. Foal shedding was associated with umbilical infection on admission (*P* = 0.016) and with ampicillin treatment during hospitalization (*p* = 0.011), and was independent of the mare’s shedding. The most common ESBL-E was *Escherichia coli*. During hospitalization, species diversity increased. Four foals were infected with ESBL-E strains, including umbilical infections and wounds. This study substantiates an alarming prevalence of shedding in neonatal foals, which should be further investigated in order to reduce resistance rates.

## 1. Introduction

Multidrug-resistant (MDR) *Enterobacteriaceae* are becoming a major worldwide concern in veterinary medicine, mainly due to the production of extended-spectrum β-lactamases (ESBLs) [1]. These widespread enzymes confer resistance to all the extended-spectrum cephalosporins and aztreonam, but not to cephamycins or carbapenems, and are usually inhibited by β-lactamase inhibitors [2]. ESBL genes are mainly plasmid-encoded and may co-carry additional antimicrobial resistances, including aminoglycosides, sulfa-derivatives, trimethoprim and quinolone resistance [3]. Therefore, treatment options of infections caused by ESBL-producing bacteria are limited. In human medicine, ESBL-E infection is associated with increased morbidity, mortality, length of hospital stay, delay of targeted appropriate treatment and higher costs [4,5]. Moreover, ESBL-E colonization has been identified as a risk factor for ESBL-E infection [6].

In horses, penicillin and cephalosporins are commonly prescribed [1], and antimicrobial resistance is of concern in a wide range of equine pathogens, including *Enterobacteriaceae* [7]. Reports of wounds, as well as respiratory and urinary tract infections, caused by ESBL-E are increasing in equine clinics [8,9]. Neonatal foals are considered as high-risk population due to their high-susceptibility and incomplete maturity of their immune system [10]. Although antibiotic resistance patterns in neonatal foals have been described [11], data on ESBL-E colonization and infections is still scarce. The motivation for this prospective study was the increasing prevalence of antibiotic resistant Gram-negative bacterial infections in hospitalized foals and the necessity to understand their origin. We hypothesized that foal ESBL-E shedding would be associated with mare shedding, as well as with specific clinical presentations and prior antibiotic treatment. We aimed to determine shedding prevalence among foal and mare pairs admitted to our hospital, to elucidate risk factors, and to determine clinical consequences.

## 2. Materials and Methods

### 2.1. Equine Study Population, Study Design and Sampling Methods

This prospective study was performed in the KSVM-VTH (Koret School of Veterinary Medicine—Veterinary Teaching Hospital). The study was approved by the Internal Research Review Committee of the KSVM-VTH (Reference number: KSVM-VTH/15_2015). Rectal swabs were collected from mares and their neonatal foals (pairs) on admission to the hospital over the course of one foaling season (November 2015–June 2016) [12]. Pairs of mares with foals under 30 days of age were included. Rectal sampling was performed immediately upon admission, prior to any medical treatment in the hospital. Rectal swabs were collected with owner consent. When both mare and foal survived and were not discharged, a second sample was taken on day 3 post admission. Overall, 55 pairs were sampled on admission, of which 33 (60%) pairs were re-sampled. When infection was detected clinically, as in umbilical infections for example, clinical samples were collected from the infection sites and *Enterobacteriaceae* isolates were tested for ESBL production. Sepsis was defined as a sepsis score greater than 11 [13], and umbilical infection was based on ultrasound and gross appearance [14].

### 2.2. Demographic and Medical Data

Medical records were reviewed for the following information: signalment (age, sex and breed) of mares and foals, parity, weight of foals, white blood cell count on admission, clinical signs on admission and during hospitalization, antibiotic therapy before and during hospitalization, surgical procedures, hospitalization length, short-term outcome and re-hospitalization.

### 2.3. ESBL-Producing Enterobacteriaceae (ESBL-E) Isolation and Species Identification

Rectal specimens were collected using bacteriological swabs (Meus s.r.l., Piove di Sacco, Italy) and were inoculated directly into a Luria Bertoni infusion enrichment broth (Hy-Labs, Rehovot, Israel) to increase sensitivity of ESBL-E detection [15]. After incubation at 37 °C (18–24 h), enriched samples were plated onto Chromagar ESBL plates (Hy-Labs, Rehovot, Israel), at 37 °C for 24 h. Pure isolates were stored at −80 °C for further analysis.

All isolates, from both rectal and clinical samples, were subjected to Vitek-2 (BioMérieux, Inc., Marcy-l’Etoile, France) for species identification and antibiotic susceptibility testing (AST-N270 Vitek 2 card). Chloramphenicol and doxycycline susceptibilities were analyzed using disc diffusion assay (Oxoid, Basingstoke, UK). ESBL-production was confirmed by combination disk diffusion using cefotaxime and ceftazidime discs (Oxoid, Basingstoke, UK), as well as cefotaxime and ceftazidime with clavulanic acid (Sensi-Discs BD, Breda, the Netherlands). Results were interpreted according to the Clinical and Laboratory Standards Institute (CLSI) guidelines [16]. Multidrug-resistant bacteria were defined as such due to their in vitro resistance to 3 or more classes of antimicrobial agents [17].

### 2.4. Molecular Characterization of ESBL-E

Isolates were examined for presence of the blaCTX-M group using a multiplex polymerase chain reaction (PCR) from ESBL-E DNA lysates, as previously described [18]. Isolates that were found to be blaCTX-M PCR negative were further examined for blaOXA-1, blaOXA2, blaOXA10 [19], blaTEM and blaSHV groups [20]. ESBL-producing *Escherichia coli* isolates were subjected to PCR in order to determine the presence of the pandemic *E. coli* ST131 strain [21]. All clinical isolates, as well as the additional fecal isolates from the same foals and their mares, were genotyped using an enterobacterial repetitive intergenic consensus (ERIC) PCR amplification using the following primer: 5′– AAGTAAGTGACTGGGGTGAGCG – 3′ [22]. Results were analyzed using GelJ software [23], and representative strains of each ERIC type were further analyzed by MLST for *E. coli*, *Klebsiella pneumonia* and *Klebsiella oxytoca*, as described before [24,25,26].

### 2.5. Statistical Analysis

Descriptive statistics were used to describe the shedding rates on admission and during hospitalization. Confidence intervals (95%) were calculated by Fisher’s (WinPEPI 11.15 Describe A). Risk assessment was performed using Chi square or Fisher’s exact tests for association between individual variables and shedding. Distribution of continuous parameters was evaluated by Shapiro-Wilk test and all were subsequently analyzed for statistical significance between two groups by Mann-Whitney U test due to non-parametric distribution. The agreement between mare and foal shedding status was analyzed by Cohen’s kappa for agreement beyond chance using the on-line Vassarstat Kappa Calculator (http://vassarstats.net/kappa.html). The McNemar test was used to examine the significance of matched pairs (shedding on admission and during hospitalization). *p* < 0.05 was considered statistically significant. A logistic regression model (multivariable analysis) was conducted using variables with *p* < 0.10, using the ENTER method (IBM SPSS Statistics 23).

## 3. Results

### 3.1. Characterization of Equine Study Population

The 55 mare–foal pairs included in this study represent 83% of the total number of mare–foal pairs admitted to the large animal department during the study period (other pairs were not sampled for logistical reasons). Out of nine pairs that were re-hospitalized, two were re-sampled on second admission, due to suspected infections (a dermal abscess in foal #1 and an umbilical infection in foal #2). Out of the sampled foals, n = 41 (74.5%) were fillies. Horses represented diverse breeds (Supplementary Information Table S1), including 60% Arabian horse (n = 66), 14.5% Tennessee walker (n = 16), 13.6% Quarter Horse (n = 15), 3.6% Single Footing (n = 4), 2.7% Appaloosa (n = 3), as well as one pair each of Missouri Fox Trotter, Friesian and Miniature breeds. Mares’ median age was 6 years, and foals’ median age was 3 days. Signalment was not consistent with the general hospital population, as these mares represent the brood mare population, along with their foals.

Admission to the hospital was due to disease of the foal, in most instances (n = 53/55, 96%). Two foals (n = 2/55, 4%) were healthy and were referred to the hospital due to disease of the mare. The pathologies were diverse (Table 1). One third of foals (n = 18/55, 33%) received antibiotics prior to hospital admission. As for the mares, 87% (n = 48/55) were healthy on arrival. One mare (n = 1/55, 1.8%) received antibiotics prior to hospitalization. For 43% of mares (n = 24/55), this was the first parturition.

### 3.2. Hospital Procedures, Antibiotic Therapy and Outcome

During hospitalization, 95% of foals (n = 52/55) and 5% of mares (n = 3/55) were treated with antibiotics. Empirical antibiotic treatment of the foals included a combination of ampicillin and amikacin [11,27] for broad-spectrum coverage. Forty percent (n = 22/55) of foals underwent a surgical procedure and 25% (n = 14/55) had a urinary catheter inserted during hospitalization. Two thirds of foals (n = 36/55, 66%) were discharged, 27% (n = 15/55) died or were euthanized, and 7% (n = 4/55) were discharged contrary to medical advice. The median hospitalization duration was 3 days (range, 0–32 days). Nine foals (25%) that were discharged were re-admitted to the hospital within one month of discharge.

### 3.3. Prevalence of ESBL-E Shedding among Foals and Mares

Shedding rates of ESBL-E in foals and mares on admission were 33% (95% CI 21–47%) and 16% (95% CI 8–29%), respectively, and were not different (*p* = 0.075). Most of the shedding foals (n = 13/18) upon admission were accompanied by a non-shedding mare. In both populations, shedding rates increased significantly during hospitalization from 33% to 85% (95% CI 70–94%) in foals and from 16% to 58% (95% CI 40–73%) in mares (Table 2). The difference in shedding rates between mares and foals during hospitalization was significant (*p* = 0.028). Nineteen out of 22 non-shedding foals on admission (86%) were re-sampled and acquired ESBL-E during hospitalization. Ten of 18 shedding foals on admission (56%) remained hospitalized and were re-sampled. Nine of them (90%) remained positive and one turned negative. As for mares, five of nine that shed on admission remained hospitalized and remained positive during hospitalization. Fourteen mares, out of 28 negative mares on admission that were re-sampled, acquired ESBL-E (50%). Therefore, shedding rates increased significantly during hospitalization in both mares and foals (*p* < 0.01 for both populations).

### 3.4. Species Distribution of ESBL-E Shedding Isolates

**Figure 1:**
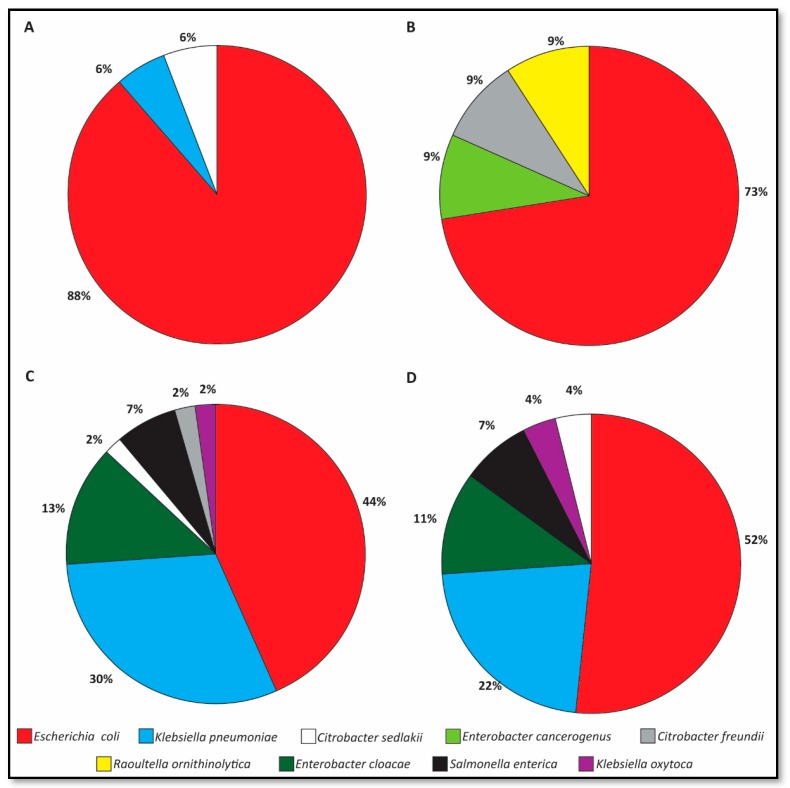
Species distribution of ESBL-E shedding on admission; foals (**A**, n = 18 isolates) and mares (**B**, n = 12 isolates); and 72 h post admission, foals (**C**, n = 46 isolates) and mares (**D**, n = 27 isolates).

Overall, 127 ESBL-E bacterial isolates were analyzed (Supplementary Information Table S2). The major fecal bacterial species on admission was *E. coli* (88% and 73% in foals and mares, respectively, Figure 1A,B). During hospitalization, the diversity of ESBL-E species increased in both populations (Figure 1C,D), with the following species distribution in foals and mares: *E. coli—*44% and 52%, respectively; *Klebsiella pneumoniae—*30% and 22%, respectively; and *Enterobacter cloacae—*13% and 11%, respectively. During hospitalization, ESBL-*Salmonella enterica* isolates were identified, consisting of 7% (3/46) and 7% (2/27) of ESBL-E shed by foals and mares, respectively. In addition, 25% (5/20) and 29% (4/14) of foals and mares, respectively, that shed ESBL-*E. coli* during hospitalization initially shed the same species on admission.

The main *bla*ESBL gene group identified in ESBL-E isolates was CTX-M-1 (Table 2). Molecular screening for the presence of pandemic *E. coli* sequence type ST131 genetic lineage among all ESBL-*E. coli* isolates (n = 66) was negative.

### 3.5. Antibiotic Susceptibility Profiles of ESBL-E Fecal Isolates

#### 3.5.1. ESBL-E Isolates on Admission

**Figure 2:**
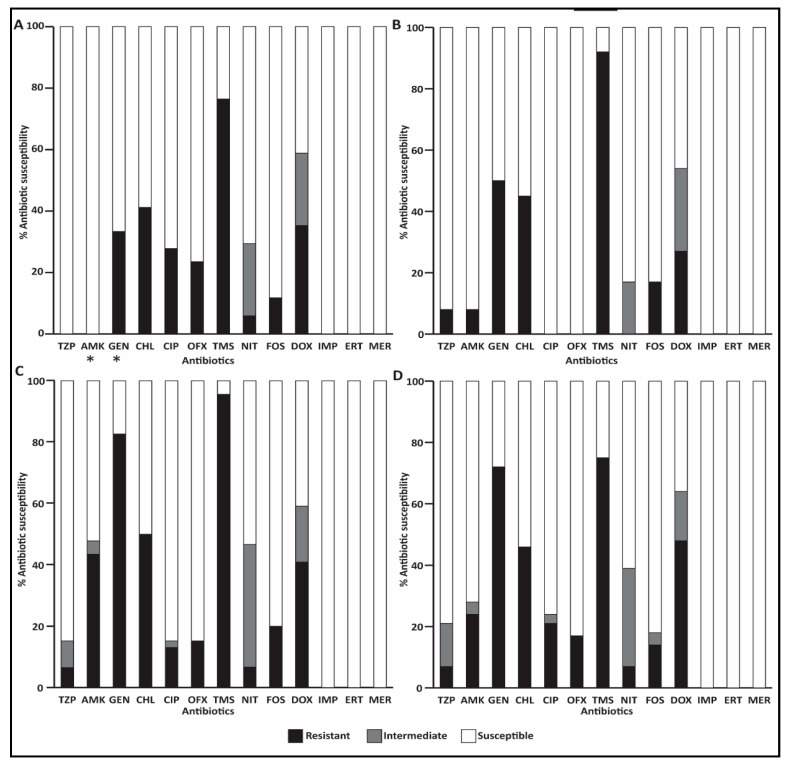
Antibiotic susceptibility profiles of ESBL-E shed on admission; foals (**A**, n = 18 isolates) and mares (**B**, n = 11 isolates); and 72 h post admission, foals (**C**, n = 46 isolates) and mares (**D**, n = 29 isolates). Significant changes are marked with asterisks.

Antibiotic resistance rates within foal and mare populations varied, mainly with respect to ciprofloxacin, ofloxacin (27% and 25% in foals vs. 0% in mares), amikacin (0% in foals vs. 8% in mares) and gentamicin (33% in foals vs. 50% in mares), with significantly higher resistance rates to gentamicin compared to amikacin in foals (*p* < 0.05) (Figure 2A,B).

#### 3.5.2. ESBL-E Isolates during Hospitalization

ESBL-E isolates recovered from hospitalized animals showed higher resistance rates compared to isolates on admission. Significant increase in resistance rates against amikacin and gentamicin were detected in strains isolated from foals (*p* < 0.05). All isolates were susceptible to carbapenems (Figure 2C,D).

#### 3.5.3. ESBL-E Isolates from Clinical Samples of Infection Sites

A total of 24 clinical samples were collected from 18 foals (6 foals had two clinical samples). Clinical samples were obtained aseptically from 10 joints, 11 umbilical samples (during omphalectomy), two wounds and one tendon sheath. Four out of 18 foals with any clinical infection (22%) had an infection with an ESBL-E strain. Overall, eight ESBL-E clinical isolates were recovered (Table 3). Five of them (63%) were identified as *E. coli*, two as *K. pneumoniae* and one as *S. enterica* (Table 3). Along with ESBL-producing *E. coli*, an MDR *Acinetobacter baumannii* strain was recovered from a wound infection sampled from the leg of foal #2. All foals with clinical infection with ESBL-E shed at least one ESBL-E, either on admission or 72-hours post admission. In two of the foals (#1 and #3) the same ESBL-E strain that was isolated from the clinical sample was also found in the fecal sample (Table 3).

All ESBL-E from clinical isolates in the study were MDR, as they also showed resistance to aminoglycosides and trimethoprim-sulpha, but were all susceptible to quinolones. In seven out of eight isolates, the ESBL gene was CTX-M group 1, and in one isolate, the ESBL gene was CTX-M group 9. Susceptibility profiles of individual isolates are displayed in Supplementary Information Table S1.

### 3.6. Risk Factor Analysis for ESBL-E Shedding

Cohen’s Kappa for the agreement beyond chance between the ESBL-E shedding status of the foal and that of the mare was 0.2894 (95% CI 0–0.5961) on admission and was 0.2546 (95% CI 0–0.6143) during hospitalization. Both results are interpreted as minimal agreement [28]. On admission, a significant association was identified between foal’s shedding and umbilical infection on admission (*p* = 0.016, 8 foals suffered from umbilical infection, out of 18 shedding foals). Odds ratio for umbilical infection in shedding foals on admission was 5.5 (95% CI 1.21–18.97). All other associations resulted in *p* > 0.10 (Supplementary Information Table S3); therefore, multivariable analysis for foal’s shedding on admission was not conducted.

During hospitalization, foal’s shedding was significantly associated with ampicillin treatment (*p* = 0.002, 26 foals were treated, out of 28 shedding foals). In a multivariate analysis for foals’ shedding during hospitalization, the model included the following parameters (*p* < 0.10): hyperthermia on arrival, ampicillin treatment during hospitalization, diarrhea during hospitalization, and length of stay. The only significant risk factor was ampicillin treatment during hospitalization (*p* = 0.011, OR = 36.88, 95% CI 2.25–603.26).

## 4. Discussion

This is the first study to investigate shedding and infection with ESBL-E in hospitalized foals and to identify the risk factors involved. Shedding rates found on admission in foals and mares were high (33% and 16%, respectively) and increased significantly during hospitalization (85% and 58% in foals and mares, respectively). In addition to identifying an alarming prevalence and incidence, neonatal foals’ ESBL-E shedding was associated with umbilical infection on admission and with ampicillin treatment during hospitalization.

Previous reports on ESBL-producing *E. coli* shedding/colonization rates in adult horses in the community ranged between 6.3 and 9% [8,29], and were lower than 10% on admission to a hospital [30]. Overall, data regarding shedding/colonization of ESBL-E in equine populations is limited and describes only ESBL-producing *E. coli*. Therefore, it is possible that the prevalence of all ESBL-E is higher, although in this study, *E. coli* was the main pathogen. ESBL-E is known to be endemic to Israel in human medicine [31], and therefore, a higher prevalence of ESBL-E circulating in the community setting may also explain the high rate on admission to the hospital. In a recent study, a high shedding/colonization rate (23.7%) was found in cattle in 40 farms in Israel. The mean prevalence of ESBL-E shedding/colonization was the highest in calves and gradually declined with maturation in adult cows [32]. In different countries, equine shedding/colonization rates may be different and generalizing our results should be done with caution.

The significant increase in shedding rates during hospitalization is alarming. Our findings support previous studies that describe a significant increase in prevalence during hospitalization [33], which was suggested to be associated with hospitalization length, mixed-purpose hospitalization yards [30] and high antimicrobial usage even in untreated animals [29]. This may explain the increase in mare ESBL-E shedding status, although they were mainly healthy. Alongside an increase in ESBL-E shedding rates, differences were observed in ESBL-E species distribution and in antibiotic resistance profiles of ESBL-E recovered from hospitalized foals and mares (Figure 1 and Figure 2). This finding supports nosocomial acquisition of ESBL-E from the hospital environment, acquisition of ESBL-encoding mobile genetic elements from other bacteria present in the gastrointestinal microbiota or due to treatment with antimicrobial drugs, as was suggested previously [30,32].

In this study, *S. enterica* was found, comprising 7% of all ESBL-E isolates in hospitalized mares and foals, and was the causative pathogen of an umbilical infection in one foal. This pathogen is highly concerning, mainly due to its zoonotic and outbreak potential. In the KSVM-VTH, all horses that are admitted with acute diarrhea are routinely isolated, and every horse that develops acute diarrhea during hospitalization is moved to the isolation ward immediately. In previous reports, different serotypes of the *Salmonella* genus were isolated from foals and identified as a cause of diarrhea and septic arthritis [34,35]. In addition, foals with gastrointestinal tract disease were 3.27 times as likely to be shedding *Salmonella* organisms compared to adult horses [36]. However, reports on ESBL-producing *Salmonella* species in neonatal foals is lacking, posing the need for further studies and the necessity of surveillance actions, including the implementation of extensive infection control measures in order to identify hot spots for acquisition.

As opposed to human newborns [37] and piglets [38], shedding of ESBL-E among foals upon hospital admission in our study was weakly associated with the ESBL-E shedding status of their mares. This result is intriguing because mares would be expected to serve as a direct source for infection of foals either in utero or during parturition, as reported in the case of women and neonates [39]. This does not seem to be the case in these mares and foals, although the wide confidence interval due to the small sample size must be noted. This finding could be related to, and affected by, differences in immunity between neonatal foals and mares and due to their high exposure to the environment in the stable. The findings of this study, supported by a previous report regarding ESBL-producing *E. coli* isolates from an equine stable [8], suggest that the equine environment is a source for ESBL-E acquisition in the foals rather than the mare. In contrast to the weak association between mare and foal shedding status, we found identical ESBL-E fecal strains mares and foals and infected foals, supporting transmission of ESBL-E strains between mares and their foals. To further understand the epidemiology of mare and foal transmission, longitudinal studies, including larger sample sizes, need to be performed.

Although the origin of bacterial infection in foal medicine is often undefined, it may be a leading cause of sepsis and death. ESBL-E may disseminate systemically, presumably through the intestinal tract of the foal as a route of invasion. Although not directly proven, bacteria may hypothetically cross the intestinal barrier into the interstitium, lymphatics and bloodstream. Reports documenting that Gram-negative enteric bacteria are the predominant isolates from neonatal foals with sepsis [34] provide further evidence of the importance of the gastrointestinal tract as a major bacterial portal of entry. Since all foals with ESBL-E-associated infections also shed ESBL-E, often with an identical strain, the association between shedding and infection is most likely. This study was prospectively designed to investigate one foaling season, in which we found four foals infected with ESBL-E. To better understand the connection between shedding and infection, longitudinal studies encompassing larger population are required.

Another clinical implication of our study concerns antibiotic treatment during hospitalization. In hospitalized foals, we identified treatment with ampicillin as significantly associated with ESBL-E shedding. This may explain the significant difference in shedding rate between foals and mares during hospitalization, as only foals were treated with ampicillin. However, there may be numerous reasons for the difference, such as maturity of the immune system. In human neonatal intensive care units, prior antibiotic treatment, combination of ampicillin/gentamicin and cephalosporin treatment were detected as risk factors for shedding and/or infection with ESBL-E [40]. In our study, most foals were treated with combination of ampicillin/amikacin [27] with or without other antibiotics; therefore, it was impossible to distinguish the effect of ampicillin as mono-therapy. In addition, treatment with cephalosporins was relatively rare and may be the reason no significant association was found. The association with ampicillin treatment should be further studied.

The limitations of this study include small sample size and retrospective medical data collection. The sample size w41as limited due to the number of admissions in the relevant foaling season. Even though the statistical analysis did reveal significant associations, a larger sample size may have resulted in additional associations.

This study underscores the importance of applying an active surveillance policy for ESBL-E shedding in foals. As we revealed the importance of ESBL-E diagnosis, future studies should include a larger cohort and further understanding of the source of these ESBL-producing strains, both on admission and in the hospital setting.

## 5. Conclusions

The results from this study substantiate the alarming occurrence of ESBL-E in equine neonatal medicine. Our data confirm that mares and their neonatal foals may shed and be infected by ESBL-E. Further studies and active surveillance should focus on community-onset, nosocomial ESBL-E shedding, and infection in foals, describing molecular characteristics and pathogenicity of ESBL-E.

## Figures and Tables

**Table 1:** Pathologies of foals and mares on admission and during hospitalization.

Pathology	No. of Horses (%)	
	On Admission	Developed during Hospitalization
Foals	n = 55	n = 33
Diarrhea	13 (24)	4 (12)
Umbilical infection	13 (24)	0
Sepsis	12 (22)	0
Prematurity	10 (17)	0
Septic polyarthritis	9 (16)	0
Orthopedic problems (other than septic polyarthritis)	9 (15)	1 (3)
Perinatal Asphyxia Syndrome (PAS)	8 (13)	0
Respiratory problems	6 (11)	0
Colic	6 (10)	1 (3)
Injury	3 (5)	0
Neurological signs (other than PAS)	1 (2)	4 (12)
Uroperitoneum	1 (2)	1 (3)
Phlebitis	0	1 (3)
Uveitis	0	2 (6)
Peritonitis	0	1 (3)
Other (hernia, guttural pouch tympany and piroplasmosis)	3 (5)	0
Mares	n = 55	n = 33
Colic	2 (4)	2 (6)
Retained placenta	2 (4)	0
Injury	1 (2)	0
Orthopedic syndromes	1 (2)	0
Placentitis	1 (2)	0
Colitis	0	1 (3)

**Table 2:** Shedding rates of ESBL-E in mares and foals on admission and during hospitalization.

	on Admission ^1^			≥ 72 h of Hospitalization ^2^		
Horses	Shedding (%)	Total No. of ESBL-E Isolates	*bla*ESBL Genes (%) ^3^	(%)Shedding	Total No. of ESBL-E Isolates	*bla*ESBL Genes (%) ^3^
Foals	18/55 (33) (95% CI 21–47)	18	*Bla*CTXM-1: 14/19 (74) *Bla*CTXM-9: 2/19 (11)	28/33 (85) ^7^ (95% CI 70–94)	46 ^4^	CTX-M-1: 31/46 (67) CTX-M-2: 1/46 (2) OXA-1: 3/46 (7)
Mares	9/55 (16) (95% CI 8–29)	11^5^	*Bla*CTXM-1: 6/11 (55)	19/33 (58) ^8^ (95% CI 40–73)	27 ^6^	CTX-M-1: 16/27 (59) CTX-M-2: 1/27 (4) CTX-M-9: 2/27 (7) TEM-163: 2/27 (7)

^1^ Rectal swabs were collected immediately on admission. ^2^ A second rectal swab was collected from all foals and mares that remained hospitalized. ^3^ ESBL genes were not identified in all ESBL-E. ^4^ Ten foals shed one ESBL-E; 15 shed two ESBL-E; two shed three ESBL-E isolates. ^5^ Eight mares shed one ESBL-E and one mare shed three ESBL-E isolates. ^6^ Eleven mares shed one ESBL-E; five shed two ESBL-E; two shed three ESBL-E isolates. ^7^ Foal shedding rates increased significantly following hospitalization and were significantly higher compared to mare shedding rates during hospitalization. ^8^ Mare shedding rates increased significantly following hospitalization.

**Table 3:** ESBL-E from clinical and samples isolated from four foals during hospitalization.

Foal	Age on Admission	ESBL-E Shedding Status				Clinical ESBL-E Infection		
		1^st^ Admission	1^st^ Hospitalization	2^nd^ Admission	2^nd^ Hospitalization	ESBL-E Species	Source	Outcome
1	<12 h	Negative	*E. coli* ST88	*K. pneumoniae* ST1552	Not sampled	*K. pneumoniae* ST1552	abscess ^1^	Discharged
2	<12 h	Negative	*E. coli* ST38	*E. coli* ST86 *K. oxytoca* ST194 *S. enterica*	*Enterobacter cloacae*	*E. coli* ST746	umbilicus ^2^	Euthanized
						*E. coli* ST746	wound	
3	<12 h	Negative	*E. coli* ST746 *K. pneumoniae* ST37	No second hospitalization		*E. coli* ST746 *K. pneumoniae* ST585	umbilicus ^3^	Euthanized
						*S. enterica*		
4	17 d	*E. coli* ^4^	Discharged	No second hospitalization		*E. coli* ST69	wound ^5^	Discharged
						*E. coli* ST69	umbilicus	

^1^ Sampled on second admission, 27 days after first hospitalization. ^2^ Sampled on second admission, 6 days after first hospitalization, which prolonged 4 days. The wound developed 9 days after second admission. ^3^ Sampled following 8 hospitalization days. ^4^ The bacteria were not recovered for further analysis. ^5^ Sampled on admission. The foal suffered from infected umbilicus and wound for a week before admission.

## Supplementary Materials

The following are available online at https://www.mdpi.com/2076-2615/9/9/600/s1, Table S1: Characterization of equine study population. Table S2: Antimicrobial susceptibility profiles of individual isolates. Susceptible = 0, intermediate susceptibility = 1, resistant = 2. Empty cells mean lack of susceptibility test results due to technical reasons. Table S3: Results of univariable analysis of variables gleaned from the medical records and evaluated for association with the outcome of ESBL-E shedding status of the individual animal.
