# Supplementary material for: Extended-Spectrum β-Lactamase-Producing Enterobacteriaceae in Hospitalized Neonatal Foals: Prevalence, Risk Factors for Shedding and Association with Infection
Source: Animals (Basel). 2019 Aug 23;9(9):600. doi: 10.3390/ani9090600 (PMC6770135; doi:10.3390/ani9090600)
Supplement: Supplementary file 1 [file animals-09-00600-s001.pdf]

Supplementary Information

Table S1. Characterization of equine study population.

Characteristics.		Prevalence (%)
Foal gender	Male	14/55 (25.5)
	Female	41/55 (74.5)
Breed	Arabian horse	66/110 (60)
	Tennessee Walker	16/110 (14.54)
	Quarter Horse	15/110 (13.6)
	Single Footing horse	4/110 (3.63)
	Missouri Fox Trotter	2/110 (1.82)
	Appaloosa	3/110 (2.7)
	Friesian	2/110 (1.82)
	Miniature	2/110 (1.82)

Table S2. Antimicrobial susceptibility profiles of individual isolates. Susceptible = 0, intermediate susceptibility = 1, resistant = 2. Empty cells mean lack of susceptibility test results due to technical reasons.

num	Isolate	Origin	Bacterial ID	TZP	AMK	GEN	CHL	CIP	OFX	TMS	NIT	FOS	DOX	IMP	ERT	MER
1	2.1.1	Foals on admission	*E.coli*	0	0	2	2	0	2	2	0	0	0	0	0	0
2	6.1.1		*E.coli*	0	0	0	0	0	0	0	0	0	0	0	0	0
3	153.1.1		*E.coli*	0	0	0	0	0	0	2	0	0	0	0	0	0
4	162.1.1		*E.coli*	0	0	0	0	0	0	0	0	2	2	0	0	0
5	177.1.1		*E.coli*	0	0	2	2	0	0	2	1	0	1	0	0	0
6	291.1.1		*E.coli*	0	0	0	0	0	0	2	0	0	1	0	0	0
7	299.1.1		*Citrobacter sedlakii*	0	0	0	0	0	0				0	0	0	0
8	307.1.4		*E.coli*	0	0	0	0	0	0	2	0	0	1	0	0	0
9	310.1.1		*E.coli*	0	0	2	2	2	2	2	0	0	2	0	0	0
10	319.1.1		*E.coli*	0	0	2	0	0	0	2	2	0	0	0	0	0
11	320.1.1		*Klebsiella pneumoniae*	0	0	0	2	0	0	2	1	2	2	0	0	0
12	334.1.2		*E.coli*	0	0	0	0	0	0	2	0	0	0	0	0	0
13	337.1.1		*E.coli*	0	0	0	0	0	0	2	0	0	1	0	0	0
14	343.1.1		*E.coli*	0	0	2	2	2	2	2	1	0	2	0	0	0
15	348.1.1		*E.coli*	0	0	0	0	2	2	2	0	0	0	0	0	0
16	350.1.1		*E.coli*	0	0	0		0	0	2	0	0		0	0	0
17	360.1.1		*E.coli*	0	0	0	2	2	0	0	0	0	2	0	0	0
18	361.1.1		*E.coli*	0	0	0	2	2		2	0	0	0	0	0	0
19	6.2.1	Foals ≥ 72 h of hospitalization	*Klebsiella pneumoniae*	0	0	0	0	2	0	2	1	2	2	0	0	0
20	6.2.2		*E.coli*	0	0	0	0	0	0	2	0	0	1	0	0	0
21	56.2.2		*Enterobacter cloacae*	2	1	2	2	2	2	2	1	2	2	0	0	0
22	85.2.1		*E.coli*	0	0	2	2	0	0	2	0	0	2	0	0	0
23	85.2.2		*Enterobacter cloacae*	0	0	2	2	0	0	2	0	0	2	0	0	0
24	167.2.1		*Klebsiella pneumoniae*	0	0	2	2	0	0	2	2	0	2	0	0	0
25	177.2.1		*E.coli*	0	0	2	2	0	0	2	1	0	0	0	0	0
26	177.2.2		*Enterobacter cloacae*	1	0	2	2	0	0	2	1	2	0	0	0	0
27	181.2.1		*E.coli*	0	2	2	2	0	0	2	0	0	2	0	0	0

28	181.2.2	*Klebsiella pneumoniae*	0	0	2	2	2	2	2	2	0	0	0	0	0
29	183.2.1	*Klebsiella pneumoniae*	0	0	2	2	0	0	2	2	0	2	0	0	0
30	183.2.2	*E.coli*	2	0	2	2	0	0	2	0	0	0	0	0	0
31	229.2.1	*E.coli*	0	2	2	2	0	0	2	0	0	2	0	0	0
32	229.2.2	*Salmonella enterica*	0	2	2	2	0	0	2	0	0	2	0	0	0
33	238.2.1	*Klebsiella pneumoniae*	0	0	2	0	0	0	2	1	0	0	0	0	0
34	243.2.1	*E.coli*	0	0	0	2	2	2	2	0	0	2	0	0	0
35	243.2.2	*Enterobacter cloacae*	0	0	2	2	0	0	2	0	0	2	0	0	0
36	279.2.2	*E.coli*	0	0	0	0	0	0	2	0	0	1	0	0	0
37	299.2.1	*Klebsiella oxytoca*	2	1	2	0	0	0	2	1	0	0	0	0	0
38	299.2.2	*Citrobacter freundii*	0	2	2	0	1	2	2	1	0	0	0	0	0
39	300.2.2	*Citrobacter sedlakii*	0	0	2	2	0	0				2	0	0	0
40	300.2.3	*Enterobacter cloacae*	0	0	2	2	0	0	2	1	0	2	0	0	0
41	303.2.2	*E.coli*	0	0	0	0	0	0	2	0	0	0	0	0	0
42	307.2.2	*E.coli*	0	0	0	0	0	0	2	0	0	0	0	0	0
43	308.2.1	*Enterobacter cloacae*	0	2	2	2	0	0	0	1	2	0	0	0	0
44	308.2.2	*E.coli*	0	0	0	2	2	2	2	0	0	2	0	0	0
45	312.2.1	*E.coli*	0	0	2	2	0	0	0	0	0	2	0	0	0
46	320.2.1	*E.coli*	0	2	2	0	0	0	2	0	0	1	0	0	0
47	320.2.2	*Klebsiella pneumoniae*	0	0	0	0	0	0	2	1	0	0	0	0	0
48	320.2.3	*Salmonella enterica*	0	2	2	0	0	0	2	0	0	0	0	0	0
49	322.2.1	*E.coli*	0	2	2	0	0	0	2	0	0	2	0	0	0
50	322.2.2	*Salmonella enterica*	0	2	2	0	0	0	2	0	0	0	0	0	0
51	322.2.3	*Klebsiella pneumoniae*	0	2	2	0	0	0	2	1	0	0	0	0	0

52	329.2.1		*Klebsiella pneumoniae*	0	2	2	0	0	0	2	1	0	0	0	0	0
53	329.2.2		*E.coli*	0	2	2	0	0	0	2	0	0	1	0	0	0
54	334.2.1		*E.coli*	0	0	2	0	2	2	2	1	0	1	0	0	0
55	334.2.2		*Klebsiella pneumoniae*	0	0	2	0	0	0	2	1	0	0	0	0	0
56	339.2.2		*E.coli*	0	0	2	0	0	0	2	0	0	1	0	0	0
57	339.2.3		*Klebsiella pneumoniae*	0	2	2	0	0	0	2	0	2	0	0	0	0
58	345.2.1		*E.coli*	0	2	2	0	0	0	2	0	2	0	0	0	0
59	348.2.1		*E.coli*	0	2	2		0	0	2	0	0		0	0	0
60	348.2.2		*Klebsiella pneumoniae*	0	2	2		0	0	2	1	0		0	0	0
61	349.2.1		*E.coli*	0	2	2	0	0	0	2	0	0	1	0	0	0
62	349.2.2		*Klebsiella pneumoniae*	1	2	2	2	0	0	2	1	2	1	0	0	0
63	356.2.2		*Klebsiella pneumoniae*	1	2	2	2	0	0	2	1	2	2	0	0	0
64	360.2.2		*Klebsiella pneumoniae*	1	2	2	2	0	2	2	1	2	2	0	0	0
65	3.1.1	Mares on admission	*E.coli*	0	0	2	2	0	0	2	0	0	0	0	0	0
66	176.1.1		*E.coli*	0	0	2	2	0	0	2	1	0	1	0	0	0
67	234.1.1		*Raoultella ornithinolytica*	0	2	2		0	0	2	1	2		0	0	0
68	239.1.1		*Enterobacter cancerogenus*	2	0	0	0	0	0	0	0	2	0	0	0	0
69	278.1.1		*E.coli*	0	0	0	0	0	0	2	0	0	1	0	0	0
70	290.1.1		*E.coli*	0	0	0	0	0	0	2	0	0	1	0	0	0
71	309.1.1		*E.coli*	0	0	2	2	0	0	2	0	0	2	0	0	0
72	309.1.2		*Citrobacter freundii*	0	0	2	2	0	0	2	0	0	2	0	0	0
73	309.1.3		*E.coli*	0	0	2	2	0	0	2	0	0	2	0	0	0
74	347.1.2		*E.coli*	0	0	0	0	0	0	2	0	0	0	0	0	0
75	351.1.1		*E.coli*	0	0	0	0	0	0	2	0	0	0	0	0	0
76	55.2.1	Mares ≥ 72 h of hospitalization	*E.coli*	0	0	2	2	0	0	2	0	0	2	0	0	0
77	84.2.1		*E.coli*	0	0	2	2	1	0	2	0	0	2	0	0	0
78	161.2.1		*E.coli*	1	0	2	2	2	0	2	0	0	2	0	0	0

79	161.2.2		*Enterobacter cloacae*	0	0	2	2	0	0	2	0	2	2	0	0	0
80	176.2.1		*E.coli*	0	0	2	2	0	0	2	1	0	1	0	0	0
81	228.2.1		*Klebsiella pneumoniae*	0	0	2	0	0	0	0	1	0	2	0	0	0
82	242.2.1		*E.coli*	0	0	0	0	2	2	2	0	0	0	0	0	0
83	278.2.1		*E.coli*	0	0	0	0	0	0	2	0	0	2	0	0	0
84	278.2.4		*Klebsiella pneumoniae*	0	0	2	0	0	0	0	2	0	1	0	0	0
85	298.2.1		*E.coli*	0	0	0	0	2	2	0	1	0	0	0	0	0
86	301.2.2		*Citrobacter sedlakii*	0	0	2	2	0	0				2	0	0	0
87	301.2.3		*Enterobacter cloacae*	0	0	2	0	0	0	2	0	0	0	0	0	0
88	302.2.1		*Salmonella enterica*	0	2	2	0	0	0	2	0	0	0	0	0	0
89	302.2.2		*E.coli*	0	0	0	0	0	0	2	0	0	0	0	0	0
90	302.2.3		*Klebsiella oxytoca*	1	1	2	0	0	0	2	1	0	2	0	0	0
91	306.2.1		*Klebsiella pneumoniae*	0	0	2	0	0	0	0	1	2	1	0	0	0
92	306.2.2		*E.coli*	0	0	0	2	0	0	2	0	0	2	0	0	0
93	309.2.2		*E.coli*	0	0	2	2	0	0	2	0	0	2	0	0	0
94	336.2.1		*E.coli*	0	0	2	0	0	0	2	0	0	2	0	0	0
95	338.2.2		*Klebsiella pneumoniae*	1	2	2	0	0	0	2	1	2		0	0	0
96	347.2.1		*E.coli*	0	2	2	0	0	0	2	0	0	1	0	0	0
97	347.2.2		*Salmonella enterica*	0	2	2	2	0	0	2	0	0	2	0	0	0
98	347.2.3		*Klebsiella pneumoniae*	1	2	2	2	0	0	2	1	0	0	0	0	0
99	357.2.3		*E.coli*	0	0	2	2	0	0	2	0	0	0	0	0	0
10	358.2.3		*Enterobacter cloacae*	0	0	2	2	0	0	2	1	0	0	0	0	0
111	359.2.1		*E.coli*	0	0	0	0	2	2	0	0	0	0	0	0	0
112	359.2.2		*Klebsiella pneumoniae*	0	2	2		2	2	2	1	2		0	0	0
113	279.6.1	Foal #1 abscess	*Klebsiella pneumoniae*	0	0	2	0	0	0	0	1	0	2	0	0	0

114	303.6.1	Foal #2 umbilicus	*E.coli*	0	2	2	0	0	0	2	0	0	2	0	0	0
115	303.7.2	Foal #2 wound	*E.coli*	0	2	2	0	0	0	2	0	0	2	0	0	0
116	339.3.1	Foal #3 umbilicus	*E.coli*	0	2	2		0	0	2	0	0		0	0	0
117	339.3.2		*Klebsiella pneumoniae*	0	2	2		0	0	2	1	0		0	0	0
118	339.3.3		*Salmonella enterica*	0	2	2		0	0	2	0	0		0	0	0
119	350.3.1	Foal#4 umbilicus	*E.coli*	0	0	0		0	0	2	0	0		0	0	0
120	350.3.3.1	Foal#4 wound	*E.coli*	0	0	0	0	0	0	2	0	0	0	0	0	0
121	278.4.1	Mare#1 2nd hospitalization, on admission	*E.coli*	0	0	0		0	0	2	0	0		0	0	0
122	279.4.1	Foal#1 2nd hospitalization, on admission	*Klebsiella pneumoniae*	0	0	2	0	0	0	0	1	0	2	0	0	0
123	302.5.1	Mare#2 2nd hospitalization, ≥ 72h of hospitalization	*E.coli*	2	0	0	2		2	2	0			0	0	0
124	303.4.1	Foal#2 2nd hospitalization, on admission	*E.coli*	0	0	2	0	0	0	2	0	0	0	0	0	0
125	303.4.2		*Klebsiella oxytoca*	2	1	2	0	0	0	2	1	0	0	0	0	0
126	303.4.3		*Salmonella enterica*	0	2	2	2	0	0	2	0	0	2	0	0	0
127	303.5.3	Foal#2 2nd hospitalization, ≥ 72 h of hospitalization	*Enterobacter cloacae*	1	2	2		0	0	2	1	2		0	0	0

Table S3. Results of univariable analysis of variables gleaned from the medical records and evaluated for association with the outcome of ESBL-E shedding status of the individual animal.

**Population Studied**	**Variable**	**Classification**	***p* value**
**Foal shedding on admission**	Breed	Quarter Horse<br>Arabian horse<br>Missouri Fox Trotter<br>Tennessee Walker<br>Appaloosa<br>Single Footing horse<br>Friesian<br>Miniature	0.755
	Gender	Male /female	0.51
	Number of pregnancy	Ranged from 1–10	0.489
	Age of mare	Ranged from 3–15 years	0.952
	Age of foal on admission	Ranged from 2 hours to 30 days	0.157
	Weight of foal on admission	Ranged from 9–59 kg	0.521
	White blood cell count on admission	Ranged from 0–43 X10^3/μL	0.464
	Clinical signs on admission	Diarrhea	0.314
		Umbilical infection	0.016
		hyperthemia	1
		Sepsis	0.167
		Prematurity	0.47
		Septic polyarthritis	0.135
		Orthopedic problems (other than septic polyarthritis)	1
		Perinatal Asphyxia Syndrome (PAS)	0.25

	Respiratory problems	0.388
	Colic	0.651
	Injury	1
	Neurological signs (other than PAS)	1
	Uroperitoneum	0.327
	Hernia	0.333
	Guttural pouch tympany	1
	Piroplasmosis	1
Clinical signs developed during hospitalization	Diarrhea	0.59
	Orthopedic problems (other than septic polyarthritis)	0.327
	Colic	1
	Neurological signs (other than PAS)	0.291
	Uroperitoneum	1
	Phlebitis	1
	Uveitis	1
	Peritonitis	0.327
Length of hospitalization	Ranged from 1–32 days	0.878
Antibiotic treatment before admission	Yes/no	0.503
	Trimethoprim– sulpha	0.327
	Ampicillin	0.543
	Penicillin	0.543
	Ceftifur	0.327
	Cefquinome	1
	Gentamicin	1
	Marbofloxacin	1
	Oxytetracycline	0.247
Surgery during hospitalization	Yes/no	0.144
Short term outcome	Died/discharged	0.529
ESBL-E shedding during hospitalization	Yes/no	1

	Breed	Quarter Horse<br>Arabian horse<br>Missouri Fox Trotter<br>Tennessee Walker<br>Appaloosa<br>Single Footing horse<br>Friesian<br>Miniature	0.249
	Gender	Male /female	0.29
	Number of pregnancy	Ranged from 1–10	0.594
	Age of mare	Ranged from 3–15 years	0.437
	Age of foal on admission	Ranged from 2 hours to 30 days	0.725
	Weight of foal on admission	Ranged from 9–59 kg	0.498
**Foal shedding ≥ 72 h of hospitalization**	White blood cell count on admission	Ranged from 0–43 X10^3/μL	0.245
	Clinical signs on admission	Diarrhea	0.574
		Umbilical infection	0.578
		hyperthermia	0.099
		Sepsis	1
		Prematurity	0.569
		Septic polyarthritis	0.569
		Orthopedic problems (other than septic polyarthritis)	0.302
		Perinatal Asphyxia Syndrome (PAS)	0.559
		Respiratory problems	1
		Colic	1
		Injury	0.284
		Neurological signs (other than PAS)	0.156

		Uroperitoneum	1
	Clinical signs developed during hospitalization	Diarrhea<br>Orthopedic problems (other than septic polyarthritis)<br>Colic<br>Neurological signs (other than PAS)<br>Phlebitis<br>Uveitis<br>Peritonitis	0.099<br>1<br>1<br>1<br>0.284<br>0.284<br>0.284
	Length of hospitalization	Ranged from 1–32 days	0.083
	Antibiotic treatment during hospitalization	Yes/no<br>Ampicillin<br>Ceftifur<br>Ceftriaxone<br>Amikacin<br>Oxytetracycline<br>Doxycycline<br>Metronidazole<br>Chloramphenicol	0.576<br>0.002<br>0.216<br>0.284<br>0.559<br>1<br>1<br>0.569<br>0.284
	Surgery during hospitalization	Yes/no	0.36
	Urine catheter placed during hospitalization	Yes/no	1
	Short term outcome	Died/discharged	1
	Re-hospitalization	Yes/no	1
**Mare shedding on admission**	Breed	Quarter Horse<br>Arabian<br>Missouri Fox Trotter<br>Tennessee Walker<br>Appaloosa<br>Single Footing horse<br>Friesian<br>Miniature	0.878

	Number of pregnancy	Ranged from 1–10	0.816
	Age of mare	Ranged from 3–15 years	0.831
	Clinical signs on admission	Colic	1
		Retained placenta	1
		Injury	0.145
		Dystocia	1
		Placentitis	1
	Clinical signs during hospitalization	Colic<br>Colitis	1
	Antibiotic treatment before admission	Yes/no	0.196
		Trimethoprim–sulpha	1
**Mare shedding during hospitalization**	Breed	Quarter Horse<br>Arabian horse<br>Missouri Fox Trotter<br>Tennessee Walker<br>Appaloosa<br>Single Footing horse<br>Friesian<br>Miniature	0.74
	Number of pregnancy	Ranged from 1–10	0.864
	Age of mare	Ranged from 3–15 years	0.67
	Clinical signs on admission	Colic	0.496
		Retained placenta	1
		Injury	1
		Dystocia	1
	Clinical signs during hospitalization	Colic	0.238
		Colitis	1
	Antibiotic treatment during hospitalization	Yes/no	1
		Penicillin	1
		Gentamicin	1
